# Casting Into The Future: Effectiveness of a 3D-Printed Fishhook Removal Task Trainer

**DOI:** 10.7759/cureus.22609

**Published:** 2022-02-25

**Authors:** Craig S Campbell, Chris Patey, Adam Dubrowski, Paul Norman, Michael Bartellas

**Affiliations:** 1 Emergency Medicine, Memorial University of Newfoundland, St. John's, CAN; 2 Emergency Medicine, Carbonear General Hospital, Carbonear, CAN; 3 Health Sciences, Ontario Tech University, Oshawa, CAN; 4 Otolaryngology, University of Ottawa, Ottawa, CAN

**Keywords:** three-dimensional (3d) printing, rural family medicine, rural emergency medicine, family medicine, emergency medicine, simulation based medical education, medical simulation, medical education, fishhook removal, fishhook injury

## Abstract

While participation in both recreational and commercial fisheries is common, it is not risk-free. Puncture wounds caused by fishhooks are commonly incurred by people who fish recreationally and commercially. Despite literature that details the challenges of treating fishhook injuries and specific techniques for fishhook removal, only a single publication focuses on teaching fishhook removal techniques to medical trainees and staff physicians.

The aim of this technical report is to investigate the efficacy of using a 3D-printed task trainer for simulating and teaching fishhook removal techniques. To facilitate this, the 3D-printed Fishhook Emergency Removal Simulator (FISH-ER 3D) was designed by the Memorial University of Newfoundland (MUN) MED 3D Network and satellite research partner, Carbonear Institute for Rural Reach and Innovation by the Sea (CIRRIS).

A sample of 22 medical residents and staff physicians were asked to evaluate the task trainer by way of a practical session, which was then followed by an evaluation survey. The overall realism of the 3D-printed task trainer components was ranked as “realistic” or “very realistic” by 86% of the evaluators. The majority of evaluators rated acquiring and performing various fishhook removal techniques using the simulator as “easy” or “somewhat easy”. Most evaluators found that using the task trainer increased user competence and confidence with fishhook removal techniques, and 100% of the evaluators rated the task trainer as a “very valuable” or “valuable” training tool.

The results of this report demonstrate support for the FISH-ER 3D as an efficacious simulator for building competence in fishhook removal techniques.

## Introduction

Fisheries are important recreational and commercial activities enjoyed by many people in Canada and around the world. Estimates conducted in 2010 by Fisheries and Oceans Canada projected that 3.61 million anglers spent a combined total of 43.3 million days fishing recreationally across the country. While participation in both recreational and commercial fisheries is common, it is not risk-free. Every year, patients arrive at emergency departments with injuries caused by fishhooks [1]. More broadly, the embedment of fishhooks in the skin is a common injury incurred by people who fish recreationally and commercially across the globe [2-6]. While special barbless hooks exist for conservation purposes, the most common style of fishhook is barbed and is designed to be difficult to remove. Therefore, injuries involving these can become complicated if proper techniques are not used when attempting to remove an embedded fishhook [2-6]. There is a variety of popular and scientific literature which details the unique challenges of treating fishhook injuries and specific techniques for fishhook removal. However, there is only a single publication focused on teaching fishhook removal techniques to medical residents and staff physicians using a task trainer [7]. This represents a scarcity of resources which clinicians can use to achieve competency in fishhook removal techniques prior to treating real patients. As a result, a niche exists for the development of a fishhook removal training simulation.

Simulation-based medical education (SBME) has been gaining traction among many medical specialties as a versatile teaching modality [8]. Simply put, it describes educational activities in medicine that use simulative aids to recreate clinical scenarios. The goal of SBME is to reduce errors during medical treatments. Using SBME, learners can improve their competence and performance in domains such as clinical skills and hands-on procedures without causing any distress to real patients [9]. Medical residency programs training physicians in emergency medicine, internal medicine, obstetrics and gynecology, urology, radiology, anesthesia, otolaryngology, and surgery have incorporated SBME with great success [9-15]. In fact, there seems to be a dose-response relationship in that more practice yields better technique and performance when using SBME as a teaching modality [11,12]. The emergence of 3D-printed task trainers creates further opportunities for the growth and improvement of SBME. A task trainer is a specialized simulator designed for teaching specific procedural skills. It provides the medium through which SBME can be delivered. In medical education, an example of a task trainer would be a lifelike, anatomical model used for teaching skills such as suturing or intubation. 3D-printing technology allows for the development of highly realistic task trainers at a lower price point than traditional simulators [16,17]. 3D-printed task trainers offer excellent versatility with applications ranging from teaching anatomy to undergraduate medical learners and teaching cerebral aneurysm repair to surgical residents. 

With this in mind, we sought to investigate whether a 3D-printed task trainer could be used to create a realistic simulation of fishhook removal for teaching purposes. To address this question, we developed the 3D-printed Fishhook Emergency Removal Simulator (FISH-ER 3D) to be used in SBME for teaching proper fishhook removal techniques. We recruited content experts to evaluate the efficacy of the FISH-ER 3D as an educational tool. We hypothesized that the FISH-ER 3D would be an efficacious SBME implement for teaching fishhook removal techniques to medical residents and staff physicians. 

## Technical report

**Figure 1:**
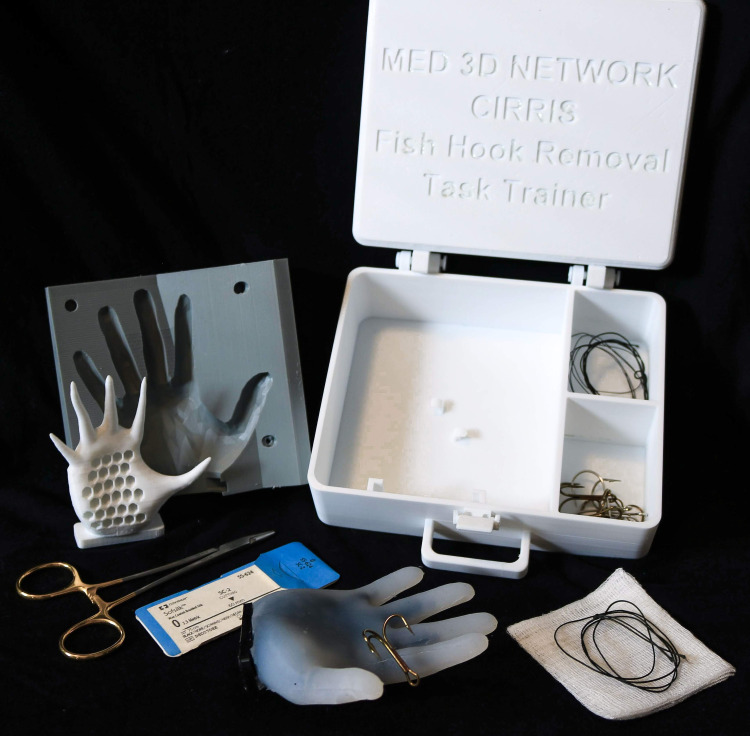
FISH-ER 3D with all necessary accessories for fishhook removal simulation Shown from back left: hand model mold and 3D-printed hand support structure before casting with silicone, carrying case, finished hand model, and first aid supplies.

The 3D-printed Fishhook Emergency Removal Simulator (FISH-ER 3D) was jointly designed by the Memorial University of Newfoundland (MUN) MED 3D Network and satellite research partner, the Carbonear Institute for Rural Reach and Innovation by the Sea (CIRRIS). CIRRIS team members were instrumental in guiding the design of prototypes, given their experience with treating fishhook injuries. Initial digital prototypes were created using Autodesk Meshmixer mesh-editing software and Autodesk Fusion 360 CAD package (Autodesk Inc., San Rafael, California). The same software was used to design the final hand model, including a hand support structure, a mold for casting silicone around the support structure, and the carrying case for transporting components of the kit. The carrying case was designed with a holder to securely house the hand model, ample space to store first-aid implements necessary for fishhook removal, and two storage compartments for safely containing fishhooks. Using this design, the FISH-ER 3D (Figure 1) can function as a completely portable, stand-alone, SBME tool for teaching various fishhook removal techniques. Following the design phase, three hand model molds, eight hand support structures, and three carrying cases were 3D-printed at the CIRRIS lab facility using an Ultimaker brand “Ultimaker 3” 3D-printer and Ultimaker Cura 3D-printing software (Ultimaker, Utrecht, Netherlands). The hand support structures are composed of polylactic acid (PLA). This material was chosen for convenience purposes. Production of a single hand support structure required 10g of PLA and a print time of two hours and 58 minutes using a 0.4mm nozzle and a layer height of 0.2mm. The total material cost was approximately $16 CAD. Once printed, the hand support structures were cast with unpigmented Smooth-On Ecoflex™ 00-30 Platinum Cure Silicone Rubber Compound. Silicone is commonly used to imitate dermal tissue in SBME because of its unique texture and durability [7,11,13]. For the FISH-ER 3D, silicone was cast to a thickness of five millimeters which simulated anatomically correct dermal depth and allowed deep embedment of fishhooks for training purposes [18]. This design feature added a life-like appearance and texture to the finished hand models.

Context

Given their clinical competence and practical experience as medical professionals, medical residents and staff physicians are well-equipped to provide thoughtful evaluations of tools designed to build proficiency in hands-on medical skills. In fact, medical residents and staff physicians are commonly sampled as content experts to evaluate 3D-printed task trainers for use in SBME [7,10-12,15,17]. For the purposes of this report, we chose to specifically sample physicians who practice family and emergency medicine. This was based on the literature, which suggests these physicians are most likely to encounter patients with fishhook injuries and must be competent in multiple techniques used for the removal of uncomplicated, embedded fishhooks [1,2,4-6]. Furthermore, because these physicians must be competent in fishhook removal, they form a representative sample of the target population who may benefit from simulation training using the FISH-ER 3D. A convenience sample of 17 medical residents and five staff physicians associated with Memorial University’s faculties of Family and Emergency Medicine, as well as the Carbonear General Hospital, was used. Workshops were held on-site at the 2019 Rural Emergency Medicine Refresher in St. John’s, the 2019 Family Medicine Academic & Wellness Resident Workshop in Bay Robert’s, and Carbonear General Hospital. All evaluators received the same instructional content and simulation opportunities.

Inputs

Simulated fishhook removal using the FISH-ER 3D included the following pieces of equipment: finished hand model, assorted fishhooks, size 1-0 braided suture, and needle drivers. Prior to interacting with the task trainer, evaluators received instruction on how to perform fishhook removal. The evaluators were given a 17-question product evaluation survey and asked to report their opinions of the task trainer (Table 1). The survey utilized Likert-type questions designed to measure ordinal scale observations and open-ended questions to gather more diverse feedback.

**Table 1:** Product evaluation survey

FISH-ER 3D fishhook emergency removal simulator						
Question #1 – realism evaluation:						
Evaluation scale: 1 = Not at all realistic, 3 = Neutral, 5 = Highly realistic, no improvements required						
Physical attributes	1	2	3		4	5
Anatomical structure						
Colour						
Shape						
Texture						
Size						
Material						
Removing fishhook						
Overall appearance						
Question #2 – Have you practiced/performed these procedural skills before (Yes or No):						
Number of times performed:						
Setting (eg. ER, outpatient clinic):						
If practiced, how often and what materials were used to simulate fishhook removal:						
For the following questions: 1 = hardest, 4 = easiest	1	2	3		4	N/A
Question #3 – Please rank how difficult each fishhook removal technique was to learn:						
Question #4 – Please rank how difficult each fishhook removal technique was to perform:						
For the following questions: 1 = least, 4 = most						
Question #5 – Please rank how much tissue damage you perceived each fishhook removal technique to cause:						
Question #6 – Please rank which fishhook removal technique you would prefer to use:						
For the following questions: 1 = least, 4 = most	1	2	3		4	N/A
Question #7 – In your opinion, how effective is the FISH-ER 3D at increasing trainees’ skills-based competency in fishhook removal?						
Question #8 – In your opinion, how effective is the FISH-ER 3D at increasing trainees’ confidence to perform fishhook removal?						
Question #9 - Please rate the value of the FISH-ER 3D as a training tool						
For the following questions, please mark Yes or No	Yes			No		
Question #10 – Did the FISH-ER 3D help you to understand something you didn’t previously understand about fishhook removal?						
Question #11 – Would you use the FISH-ER 3D to assist with your ongoing training and/ or education?						
Question #12 – Would you recommend the use of the FISH-ER 3D to assist with the training and education of other professionals and students?						
Question #13 – Would you recommend improvements to future iterations of the FISH-ER 3D?						
Question #14 – Do you find the carrying case to be a useful addition to this simulator?						
Question #15 – Would you recommend any changes to the carrying case?						
Question #16 – Please expand on any recommendations you have for future versions of this model						
Question #17 For the following question, please select the statement with which you most agree	Please indicate your selection					
The FISH-ER 3D requires extensive improvements before it can be considered for training.						
The FISH-ER 3D requires minor improvements before it can be considered for training.						
The FISH-ER 3D requires no improvements and can be used for training.						

Process

To evaluate the realism and value of the FISH-ER 3D as an educational tool, each evaluator was provided with brief written instructions developed by the research team detailing how to perform three common fishhook removal techniques: retrograde removal, string-pull removal, and advance and cut removal [2,3,6,7]. These three removal techniques were also demonstrated to the evaluators by a member of the research team (Video 1). Following this, evaluators were given five minutes to interact with the task trainer and practice the different fishhook removal techniques. Then, the evaluators were asked to complete the task trainer evaluation survey.

**Video 1:** Demonstration of fishhook removal using the FISHER-3D

The survey data are presented using descriptive statistics. The reason for presenting the data in this manner is because the aim of this report was to assess the FISH-ER 3D as a tool for teaching fishhook removal based on feedback from a relatively small group of evaluators. By using descriptive statistics, it was possible to summarize evaluators’ opinions of various aspects of the task trainer [19,20]. Key strengths and weaknesses of the FISH-ER 3D were easily interpreted using this method of analysis. Ultimately, this permitted an informed discussion regarding the efficacy of the FISH-ER 3D for teaching proper fishhook removal techniques to medical learners and staff physicians. It also clearly outlined aspects of the task trainer that would benefit from improvement in the future.

Products/outcomes

Realism

Survey analysis showed that evaluators found the overall appearance of the hand model to be realistic (Table 2). Of the seven distinct physical characteristics evaluated, anatomical structure, model shape, model texture, model size, model material, and removing fishhooks from the model were all given the rating of “Realistic” by the majority of evaluators. Only the color of the hand did not receive a rating of “Realistic”, as the majority of evaluators gave it a “Neutral” rating. These findings indicate that the FISH-ER 3D hand model serves as a realistic simulation of an actual patient’s hand. Therefore, increasing the realism of simulated fishhook removal training.

**Table 2:** Mode, frequency count, and mean ratings of hand model realism Evaluation Scale: 1 = Not realistic, 2 = Lacks realism, 3 = Neutral, 4 = Realistic, 5 = Highly realistic

QUESTIONS	TOTAL (N)	Mode (%)	Mean + SD
Anatomical structure	22	Realistic (68%)	4.2 + 0.7
Model color	22	Neutral (50%)	3.1 + 1.2
Model shape	22	Realistic (55%)	4.1 + 0.8
Model texture	22	Realistic (45%)	3.8 + 0.9
Model size	22	Realistic (45%)	3.6 + 0.9
Model material	22	Realistic (59%)	4.0 + 0.8
Removing hook from model	22	Realistic (59%)	4.1 + 0.6
Overall appearance of model	22	Realistic (59%)	4.1 + 0.6

Value - Acquisition and Performance of Fishhook Removal Techniques

**Figure 2:**
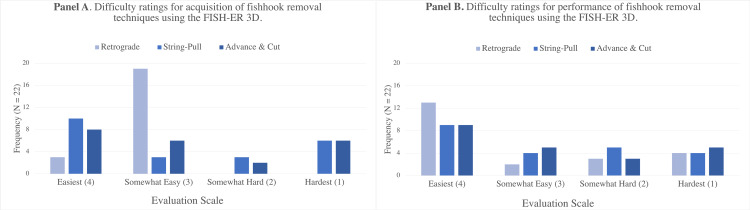
Difficulty ratings for acquisition (Panel A) and performance (Panel B) of fishhook removal techniques using the FISH-ER 3D

Each evaluator practiced three common fishhook removal techniques several times using the FISH-ER 3D. Following this, the evaluators were asked to rank how difficult it was to acquire and perform retrograde, string-pull, and advance & cut techniques for fishhook removal (Figure 2).

The survey results indicate that, generally, the evaluators found both the acquisition and performance of fishhook removal techniques to be easy. All evaluators rated the acquisition of the retrograde removal technique as “Easy” or “Somewhat Easy”, while 59% (N = 13) and 64% (N = 14) of evaluators rated both the string-pull and advance & cut methods as “Easy” or “Somewhat Easy”. The string-pull and advance & cut removal techniques may have been more challenging for evaluators to acquire, as 41% (N = 9) and 36% (N = 8) of evaluators rated these techniques as “Somewhat Hard” or “Hardest” to acquire, respectively. 68% (N = 15) of evaluators found retrograde removal “Easy” or “Somewhat Easy” to perform using the task trainer. Performance of string-pull and advance & cut removal was rated as “Easy” or “Somewhat Easy” by 59% (N = 13) and 64% (N = 14) of evaluators, respectively. There was a relatively small difference between which fishhook removal technique was most difficult to perform. The retrograde removal, advance & cut, and the string-pull methods were rated as either “Somewhat Hard” or “Hardest” by 32% (N = 7), 36% (N = 8), and 41% (N = 9) of evaluators, respectively. Given how easily the evaluators reported they were able to acquire and perform three common fishhook removal techniques after practicing with the FISH-ER 3D, it appears it is indeed valuable for simulating and building skills-based competency in fishhook removal.

Value - Impact on Learning

**Figure 3:**
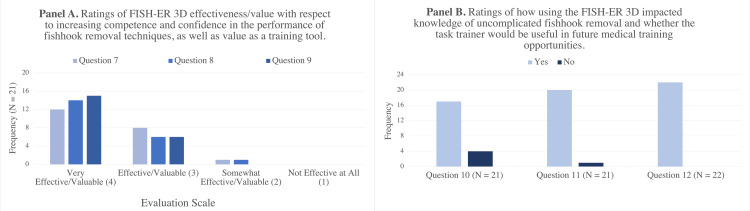
Ratings of FISH-ER 3D effectiveness/value as a training tool (Panel A) and impact on knowledge/future utility (Panel B) Question 7 - In your opinion, how effective is the FISH-ER 3D at increasing trainees’ skills-based competency in fishhook removal? Question 8 - In your opinion, how effective is the FISH-ER 3D at increasing trainees’ confidence to perform fishhook removal? Question 9 - Please rate the value of the FISH-ER 3D as a training tool. Question 10 - Did the FISH-ER 3D help you to understand something you didn’t previously understand about fishhook removal? Question 11 - Would you use the FISH-ER 3D to assist with your ongoing training and/ or education? Question 12 - Would you recommend the use of the FISH-ER 3D to assist with the training and education of other professionals and students?

In addition to rating how difficult it was to acquire and perform fishhook removal techniques, evaluators were also asked to explore how using the task trainer to practice these techniques impacted their attitudes and knowledge surrounding fishhook removal (Figure 3). Evaluator responses to this portion of the survey highlight the utility of the FISH-ER 3D as a training tool. Ninety-five percent (N = 20) of evaluators indicated that they found practicing fishhook removal techniques using the task trainer to be “Very Effective” or “Effective” at increasing competence (Question 7) and confidence (Question 8) related to fishhook removal. Just 5% (N = 1) of the evaluators indicated that using the task trainer to practice fishhook removal techniques was only “Somewhat Effective” at increasing confidence and competence related to fishhook removal. When evaluating the FISH-ER 3D’s value as a training tool for medical learners and physicians, 71% (N = 15) of the evaluators rated it as “Very Valuable”, while 29% (N = 6) rated it as a “Valuable” training tool (Figure 3, Question 9).

Furthermore, 81% (N = 17) of the evaluators found the FISH-ER 3D helpful in improving their understanding of uncomplicated fishhook removal, as opposed to 19% (N = 4) who did not (Figure 3, Question 10). The simulator received further positive reviews from evaluators, as 95% (N = 20) indicated they would incorporate the FISH-ER 3D to assist with their ongoing training and education needs (Question 11). All evaluators (100%, N = 22) reported that they would recommend the simulator to assist with the training and education of other medical learners and physicians (Question 12). This data suggests that the FISH-ER 3D is a valuable SBME tool with the ability to increase trainee confidence and competence. As well, evaluator responses suggest that the FISH-ER 3D may be a useful tool for teaching proper fishhook removal techniques to other healthcare professionals.

Value - Overall Response to the Task Trainer

**Figure 4:**
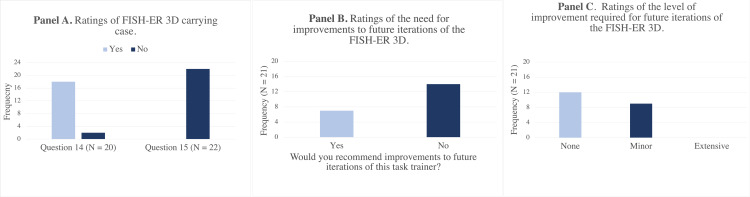
Ratings of FISH-ER 3D carrying case, need for improvements, and level of improvement required for future iterations of the model Question 14 - Do you find the carrying case to be useful addition to this simulator? Question 15 - Would you recommend any changes to the carrying case?

An important aspect of the FISH-ER 3D is portability. After interacting with the simulator, evaluators were surveyed about the accompanying carrying case to complete the evaluation of the training kit (Figure 4). The carrying case was rated as being a useful addition to the FISH-ER 3D by 90% (N =18) of the evaluators (Question 14). No evaluators recommended any changes to the carrying case (Question 15). 

Evaluators were also asked whether they would recommend any changes to future versions of the FISH-ER 3D and whether they would recommend no improvements at all, minor, or extensive improvements. Only 33% (N = 7) of evaluators indicated that they would recommend improvements to future versions of the simulator, while 67% (N = 14) of evaluators did not feel that any improvements were necessary (Figure 4). Although no evaluators found that future versions of the simulator would require extensive improvements, 43% (N = 9) reported that minor improvements should be made. At 57% (N = 12), most evaluators felt that no improvements would be required to make the FISH-ER 3D a more effective teaching tool (Figure 4). Improvements that evaluators indicated they would like to see made on future versions of this simulator revolved around the physical realism of the hand model. Evaluators commented that they would like to see a larger-sized hand model with a more realistic skin color, the addition of veins, arteries, and tendons to the hand model, and a thicker silicone sleeve surrounding the hand, which behaves more like human skin when manipulating an embedded fishhook. Overall, evaluators provided positive support for all aspects of the FISH-ER 3D with only a few minor improvements required to maximize the value of this simulator.

## Discussion

Fishhook removal is a clinical skill that theoretically lends itself well to SBME. Caring for fishhook injuries requires medical professionals to be competent in hands-on skills which incorporate coordination, dexterity, and tactile feedback [1,2,4-6,9-14,17]. Simulation has received limited use as a medium for teaching fishhook removal techniques to medical residents and staff physicians. Perhaps, this is partly due to the difficulty of acquiring realistic yet cost-friendly models for teaching and practicing hands-on skills [16]. Surprisingly, the use of 3D-printed task trainers for SBME in fishhook removal has not yet been fully explored, despite the robust capabilities of this technology. Advances in 3D-printing technology provide opportunities to readily develop products for SBME that bridge the gap between high-quality realistic task trainers and affordability. Therefore, this technical report was designed to explore whether a 3D-printed task trainer could serve as an effective tool for SBME in fishhook removal techniques. 

According to the literature, effective SBME task trainers should provide a means for trainees to acquire and perform hands-on skills in a controlled environment without the participation of real patients, as well as allow trainees to engage in repetitive practice while receiving feedback from an instructor. Additionally, the simulator should be adaptable to permit variations in clinical presentation and be a reasonable representation of the clinical scenario a trainee would be expected to encounter and manage in an actual patient care setting [9-15,17]. Based on these attributes and the findings of this report, the FISH-ER 3D is an effective tool for SBME. Careful design resulted in the production of a teaching tool that was portable enough to give the researchers complete control over the simulation environment and durable enough to allow evaluators to repeatedly practice fishhook removal techniques without compromising the physical integrity of the hand model. 

The FISH-ER 3D also demonstrated versatility. Consideration was given to producing a hand model with anatomically correct skin depth for an adult patient allowed evaluators to practice removing fishhooks that were embedded at different depths in the simulated tissue on both the palmar and dorsal surfaces of the hand and digits. This was an important feature as patients may present to real clinical settings with a fishhook embedded in their tissue at varying depths. Having realistic simulated tissue allowed multiple fishhook removal techniques to be practiced by evaluators. Based on their responses, the majority of evaluators found the FISH-ER 3D provided a physically realistic simulation of fishhook injuries that might be encountered in a real-life clinical situation. 

Most importantly, the FISH-ER 3D facilitated learning through SBME. This technical report demonstrates that different fishhook removal techniques can be easily learned and performed using the FISH-ER 3D. Every evaluator reported increased competence in fishhook removal techniques after practicing on the model. Additionally, the evaluators endorsed increased confidence in their ability to successfully remove an embedded fishhook from a real patient. In fact, the FISH-ER 3D was so well received that nearly all the evaluators indicated they would use it again for their own training needs. Every evaluator recommended it be used to assist the training of medical learners and healthcare professionals. These outcomes are extremely important as they illustrate the core value of the FISH-ER 3D as an educational tool. Therefore, confirming the hypothesis that a 3D-printed task trainer can serve as an efficacious implement for SBME in proper fishhook removal techniques. 

A limitation of this report was the reliance on survey data and the possible effect of response bias on the results. The survey section on the acquisition and performance of fishhook removal techniques may have been particularly susceptible to response bias, as the evaluators may have wanted to appear competent in fishhook removal techniques. Given their positions as medical residents and staff physicians, the evaluators may have been less willing to report an inability to competently perform hands-on medical procedures. Another limitation was the larger proportion of medical residents evaluating the model compared to the number of staff physicians. As a group, the medical residents had less experience removing embedded fishhooks than the staff physicians. However, their relative inexperience provided the researchers an understanding of how those with less practice in fishhook removal interacted with the simulator. Lastly, this report did not incorporate any comparison of the FISH-ER 3D to conventional inanimate or animal simulation tools. While it was clearly stated that this was not a focus of this report, it would have facilitated a better estimation of the value of the FISH-ER 3D.

## Conclusions

The collective findings of this report demonstrate support for the FISH-ER 3D as an efficacious SBME tool for building competence in fishhook removal techniques. The simulator received positive comments from all medical residents and staff physicians who evaluated it. Of the few improvements recommended for the FISH-ER 3D, the evaluators felt that adding veins, arteries, tendons, and thicker simulated tissue to the hand models would build upon the overall realism of the hand model and improve the quality of fishhook removal simulations. The evaluators did not identify any issues with the functionality of the simulator while they were interacting with it. In conclusion, it is apparent that 3D-printed task trainers are, in fact, effective tools for simulation-based medical education in fishhook removal techniques.

Future work with this model should focus primarily on further design improvement by incorporating changes to the FISH-ER 3D that were suggested by the evaluators. Other avenues for future investigation include an evaluation of whether learning fishhook removal techniques using the FISH-ER 3D leads to the successful removal of fishhooks on subsequent attempts. It is possible that this task trainer may also be an asset for training medical residents and staff physicians in foreign body removal for objects other than fishhooks. Additionally, exciting research opportunities exist with a head-to-head comparison of the FISH-ER 3D to high-fidelity models and animal parts used for simulation in medicine. This comparison should consider the cost, performance, durability, realism, and training outcomes associated with using each model.
